# Efficacy of a Superficial Cervical Plexus Block as an Alternative to General Anesthesia for Mandibular and Perimandibular Surgical Procedures: A Systematic Review

**DOI:** 10.7759/cureus.104095

**Published:** 2026-02-23

**Authors:** Ojasvee Hiran, Tejraj Kale, Radhika Pathak, Rahul Bichile, Kairav Vadher

**Affiliations:** 1 Oral and Maxillofacial Surgery, KLE Vishwanath Katti Institute of Dental Sciences, Belagavi, IND

**Keywords:** incision and drainage, mandibular fracture, regional anaesthesia, superficial cervical plexus anatomy, superficial cervical plexus block

## Abstract

Any oral and maxillofacial surgery often requires reliable anesthesia to ensure analgesia, patient comfort, and surgical efficiency. While general anesthesia is the traditional choice, it carries risks of cardiopulmonary complications, prolonged recovery, and higher resource utilization. A superficial cervical plexus block has emerged as a potential alternative, offering simplicity, safety, and cost-effectiveness. This systematic review aimed to evaluate the efficacy and safety of the superficial cervical plexus block as an alternative to general anesthesia in mandibular and perimandibular, oral, and maxillofacial surgical procedures.

Following the Preferred Reporting Items for Systematic Reviews and Meta-Analyses (PRISMA) 2020 guidelines, a comprehensive search of PubMed, Cochrane, Google Scholar, and Wiley (2000-2024) was conducted. Eligible studies included randomized controlled trials, clinical studies, case series, and case reports involving the superficial cervical plexus block in mandibular and perimandibular surgeries. The risk of bias was assessed using the RoB-2 tool and the Joanna Briggs Institute tools for specific types of included studies.

Nine studies (n = 164 patients) were included where the superficial cervical plexus block alone or when combined with supplemental local blocks consistently provided effective intraoperative and postoperative analgesia. In comparative trials, superficial cervical plexus block groups demonstrated significantly lower postoperative analgesic requirements and pain scores compared to general anesthesia or local infiltration (p < 0.05). Case series and reports further supported the superficial cervical plexus block’s high success rate and patient satisfaction, with negligible complications and rare conversion to general anesthesia.

The superficial cervical plexus block is a safe and effective alternative to general anesthesia in selected mandibular and perimandibular surgical procedures, particularly in high-risk or resource-limited settings. Despite heterogeneity and small sample sizes, current evidence supports its wider clinical adoption. Large multicenter randomized controlled trials and standardized protocols are recommended to strengthen the evidence base.

## Introduction and background

In order to carry out any procedure in the mandibular and perimandibular regions without pain, anesthesia is of utmost importance, which also helps in anxiety management among patients. The use of anesthesia in dental procedures dates back to 1846 in Boston, when Dr. William T.G. Morton performed the first painless dental extraction using ether. Various modes of providing anesthesia are available based on the category of procedure to be conducted [1].

Traditionally, general anesthesia (GA) has been the mainstay for achieving adequate analgesia, amnesia, and muscle relaxation in major maxillofacial procedures. However, GA carries inherent risks, especially in patients with comorbidities, and often requires more extensive perioperative care, advanced monitoring, and prolonged recovery time. In contrast, regional anesthetic techniques have garnered growing interest due to their potential advantages, including fewer systemic complications, reduced need for airway management, lower postoperative pain scores, and shorter hospital stays [2].

Among the regional techniques, the superficial cervical plexus block (SCPB) has emerged as a promising alternative. The superficial cervical plexus is formed by the ventral rami of the C2-C4 spinal nerves and emerges at the posterior border of the sternocleidomastoid muscle at the level of C3-C4 cervical vertebrae, providing sensory innervation to the anterolateral neck and lower face. The SCPB is performed by infiltrating local anesthetic along this anatomical plane, typically at the midpoint of the sternocleidomastoid's posterior border, targeting the four main terminal branches: lesser occipital, greater auricular, transverse cervical, and supraclavicular nerves. Unlike the deep cervical plexus block, which targets motor and deeper sensory structures, SCPB is a superficial technique with a favorable safety profile, avoiding risks associated with neuraxial or vascular puncture. This block can provide effective analgesia for superficial surgeries of the neck and lower face and, when combined with supplementary local infiltration or other regional blocks, may extend coverage to mandibular and perimandibular procedures [3].

SCPB has been successfully employed in a range of head and neck surgeries [4]. However, its utility in oral and maxillofacial surgery (OMFS) has only recently begun to gain attention. Preliminary studies and case reports suggest that SCPB, either alone or in combination with other local or regional blocks, can offer effective intraoperative and postoperative analgesia for procedures such as mandibular fracture fixation, submandibular gland excision, and superficial soft tissue surgeries of the face and neck [5]. The efficacy of SCPB in OMFS can be assessed by multiple parameters: quality of intraoperative analgesia, need for conversion to GA or additional sedation, patient satisfaction, postoperative pain control, complication rates, and time to recovery.

This review aims to evaluate the efficacy of the SCPB as an alternative to GA in OMFS. By aggregating findings from various studies, it will provide insights into the safety, effectiveness, and feasibility of SCPB in different clinical scenarios, thus helping clinicians make informed decisions about anesthetic planning.

The primary objectives of this review are to assess the effectiveness of SCPB in providing adequate anesthesia for OMFS procedures, to compare intraoperative and postoperative outcomes between SCPB and GA, and to evaluate patient complication rates associated with SCPB. By addressing these objectives, this review seeks to contribute to evidence-based practice in maxillofacial anesthesia, potentially broadening the use of SCPB in both routine and specialized clinical settings.

## Review

Materials and methods

Protocol and Registration

The study followed the Preferred Reporting Items for Systematic Reviews and Meta-Analyses (PRISMA) guidelines in the 2020 checklist. The review has been officially documented within PROSPERO under registration number CRD42024590655.

Eligibility Criteria

The PICO criteria for this review are presented in Table 1.

**Table 1:** PICO criteria for this review

PICO criteria	
P (participants)	Patient undergoing mandibular and perimandibular surgeries under superficial cervical plexus block between the ages of 18 and 90 years
I (intervention)	Superficial cervical plexus block for mandibular and perimandibular surgeries
C (comparison)	With or without a control group
	Comparison can be with general anesthesia
O (outcome)	Perioperative pain and perioperative complications

The eligibility criteria are presented in Table 2.

**Table 2:** Eligibility criteria SCPB: superficial cervical plexus block

Inclusion criteria	Exclusion criteria
1. Studies including oral and maxillofacial surgical procedures performed under SCPB	1. Articles not fulfilling the inclusion criteria
2. Studies published between 2000 and 2024	2. Studies done in any language other than English
3. Study design: randomized controlled trials (RCTs), non-RCTs, clinical trials, retrospective studies, case series, case reports, or any clinical cross-sectional study	3. Opinion articles, letters
4. Studies that assess preoperative pain and other complications	4. Animal studies, in vivo studies
5. Studies involving supplemental blocks like inferior alveolar nerve block, lingual nerve block, long buccal nerve block, and local infiltration	5. Studies involving deep/intermediate form of cervical plexus block

Information Source

A thorough electronic search was done using databases like PubMed, Google Scholar, Wiley Library, and Cochrane from January 1, 2000, to July 2024. Titles and abstracts were screened, and if the full text was not available for a potential study, the author was contacted for the same. Cross-references and citations from similar studies were also assessed. Studies other than English language were excluded. We resorted to manual methods for literature search when the full texts of relevant studies were not accessible via electronic databases.

Search Strategy

The keywords used for the literature search are “superficial cervical plexus block”, “SCPB”, “regional anaesthesia”, “oral and maxillofacial surgery”, “mandibular surgery”, “ mandibular fracture”, and “incision and drainage”. These keywords were clubbed, and an advanced search was done in each database using these keywords: [“SCPB” AND “mandibular surgery”], [“SCPB” and “mandibular fracture”], [“SCPB AND incision and drainage], [“regional anaesthesia” AND “maxillofacial procedures”], [“regional anaesthesia” AND “maxillofacial procedures” AND “mandibular fracture”], [“SCPB” AND “mandibular surgery” AND “condylar fracture”], and [“SCPB” AND “mandibular surgery” AND “Incision and drainage”].

Study Selection

Preliminary screening identified 55 studies from various databases, comprising 37 articles from Google Scholar, nine articles from PubMed, three articles from Wiley Library, and six articles from Cochrane, out of which 16 articles were removed as duplicates. Of the remaining 39 studies searched to screen for title and abstract, two articles could not be retrieved. Out of the remaining 37 articles, 28 were excluded, including 19 articles that did not meet the inclusion criteria, two articles written in languages other than English, one article that was a citation, and six articles that were books and literary articles. Thus, this systematic review included nine studies. Two independent reviewers screened titles and abstracts, followed by full-text articles, using a standardized screening form implemented in Microsoft Excel (Microsoft Corp., Redmond, WA, USA). Disagreements were resolved through discussion to reach consensus; a third reviewer was available for arbitration but was not required.

Data Extraction

Data screening was done by two reviewers individually. The first screening was done on the basis of the title and abstract. If enough information was not available, then full-text articles were obtained to review the studies. Upon acquiring the full texts, a comprehensive review was conducted by reading each article in its entirety to determine whether it met the inclusion criteria. Data extraction was conducted independently and in duplicate by two reviewers using a pre-piloted standardized Microsoft Excel template capturing authors' name and year (name of the first author and year of article publication), study design (randomized controlled trials (RCTs), non-RCTs, prospective clinical studies, case series, and case reports), sample size (number of patients included in the study), age (mean age group of the patients involved), procedure, intervention (SCPB along with type of local nerve block used), comparison (with or without any control group), analgesic effect (perioperative pain score using the Visual Analogue Scale (VAS)), other outcomes (complications such as nausea, vomiting, conversion to GA, and baseline vital parameters), results (results of each study), and conclusion (conclusions of each study, including limitations if mentioned). Any discrepancies were resolved by consensus between the extractors.

Risk of Bias Assessment

For RCTs, Cochrane’s RoB-2 tool was used. For every other study design, the appropriate Joanna Briggs Institute (JBI) critical appraisal tool was used.

Synthesis of Result

A meta-analysis was not performed due to significant clinical and methodological heterogeneity across studies, including variations in SCPB techniques, surgical procedures, outcome measures, and follow-up durations. Additionally, small sample sizes and limited RCTs precluded meaningful statistical pooling.

Results

Study Selection

**Figure 1:**
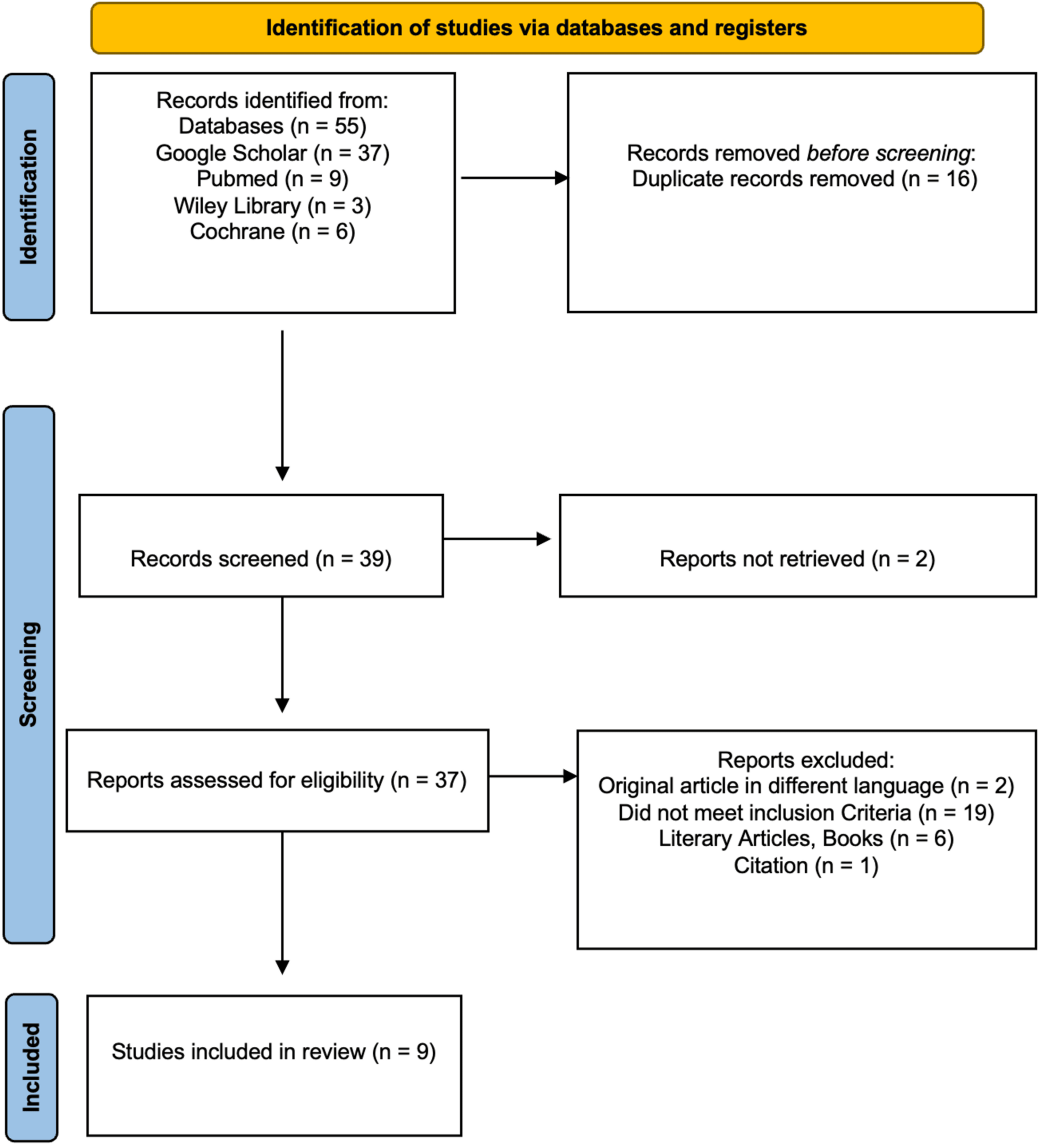
PRISMA 2020 flow diagram for study selection PRISMA: Preferred Reporting Items for Systematic Reviews and Meta-Analyses

This systematic review followed the PRISMA 2020 guidelines (Figure 1).

Study Characteristics

The characteristics of all the included studies with the above-mentioned data items have been combined in a table (Table 3).

**Table 3:** Characteristics of all included studies SCPNB: superficial cervical plexus nerve block; VAS: Visual Analogue Scale; GA: general anesthesia

Serial no.	Study-author name and year	Study design	Sample size	Procedures	Control group	Intervention	Outcome: perioperative analgesia						Outcome: complications	Conclusion
															1	Singh et al., 2020 [6]	Prospective clinical trial	24 patients: 16 females, 8 males	Incision and drainage	No control group	SCPNB with trigeminal local mandibular nerve blocks	Intraoperative VAS score: ranged from 0 to 8 with a mean of 1.5 ± 1.87						1 patient was taken up for GA due to unbearable pain	SCPNB with trigeminal nerve block is more effective in mandibular and perimandibular cases with a low rate of complications
2	Patel et al., 2023 [7]	Comparative randomized controlled trial	Group A: 30 patients; Group B: 30 patients	Procedures not specified	Group A: sedation with systemic GA	Group B: sedation with SCPNB	Intraoperative total postoperative rescue analgesic requirement in 24 hours in Group A (97.5 ± 13.75 µg and 2,566.66 ± 504 mg) and Group B (70.16 ± 13.09 µg and 833.33 ± 874.28 mg); p-value was <0.001. VAS score: 6 hours postoperatively Group A > 4 and Group B < 4 (p-value < 0.005). Postoperatively 16, 20, and 24 hours, insignificant difference between 2 groups with a p-value > 0.05						Group A: 2 patients had nausea and vomiting postoperatively; Group B: no complications were reported	It can be concluded that in balanced anesthesia technique, superficial cervical plexus nerve block gives better results in immediate postoperative analgesia and is safe with a significantly lower rate of complications as compared to GA															
																														3	Kumar et al., 2023 [1]	Prospective randomized clinical trial	Category A: 25 patients; category B: 23 patients	1. Incision and drainage; 2. cyst enucleation; 3. open reduction and internal fixation	Category A: combination of local infiltration and regional anesthesia	Category B: regional anesthesia with SCPNB	Waiting period for analgesic requirement postoperatively: Category A: 75.12 ± 14.24 minutes; Category B: 137.23 ± 32.43 minutes. p-value: 0.0312. VAS score: duration (in mins): at 15 and 30 minutes (p ≤ 0.05); at 60 and 120 minutes (p > 0.01)						No complications reported in both groups	Postoperative analgesia is better with SCPNB; increased duration for the requirement of analgesics during the recovery period
																10	15	30	60	120																								
															Category A	4	4	3	2	1																								
															Category B	4	3	2	2	1																								
															4	Kanthan, 2016 [3]	Case series	10 patients: 6 males, 4 females	1. Incision and drainage; 2. open reduction and internal fixation; 3. cyst enucleation; 4. cervical lymph node biopsy	No control group	SCPNB with supplemental nerve block	Not assessed						No complications reported	SCPNB with concomitant mandibular nerve and/or long buccal nerve block has a high success rate, low complication rate, and high patient acceptability as shown in the study															
																																													5	Hakim et al., 2019 [8]	Case series	10 patients: 6 males, 4 females	1. Incision and drainage; 2. open reduction and internal fixation; 3. cyst enucleation; 4. submandibular lymph node biopsy	No control group	SCPNB with supplemental nerve block	Not assessed						No complications reported	SCPNB with concomitant mandibular nerve and/or long buccal nerve block has a high success rate, low complication rate, and high patient acceptability as shown in the study
																																																												6	Zhao et al., 2024 [5]	Case series	7 patients: 4 females, 3 males	Excision of tumors in submandibular and sublingual regions	No control group	SCPNB with local infiltration anesthesia	Postoperative analgesic effect lasted for 6 hours						No complications were recorded	Ultrasound-guided SCPNB combined with local infiltration is a simple, safe, and effective anesthesia method for some patients with specific diseases, especially elderly patients with poor general condition who are at risk of GA
																																																																											7	Saripalli et al., 2022 [9]	Case presentation	1 male	Open reduction and internal fixation	No control group	SCPNB with supplemental nerve block	Effect of analgesia lasted from 15 mins after injection till 6 hours postoperatively						No complications	From this study, it can be concluded that mandibular fracture fixation can be performed under SCPNB and local anesthesia effectively without any complications
8	Shteif et al., 2008 [10]	Case report	3 patients: 1 male, 2 females	Incision and drainage	No control group	SCPNB with long buccal or inferior alveolar nerve block	Not assessed						No complications	SCPNB with concomitant mandibular nerve and/or long buccal nerve block has a high success rate, low complication rate, and high patient acceptance rate in cases of submandibular and submental space infection																																																																											
																																																																																										9	Peksöz et al., 2022 [11]	Case report	1 male	Lipoma excision	No control group	SCPNB nerve block	Not assessed						No complications	In the short-term surgery of soft tissue lesions in the lesser occipital nerve and great auricular nerve dermatome areas, anesthesia can be achieved through a superficial cervical plexus block

Risk of Bias in Studies

**Figure 2:**
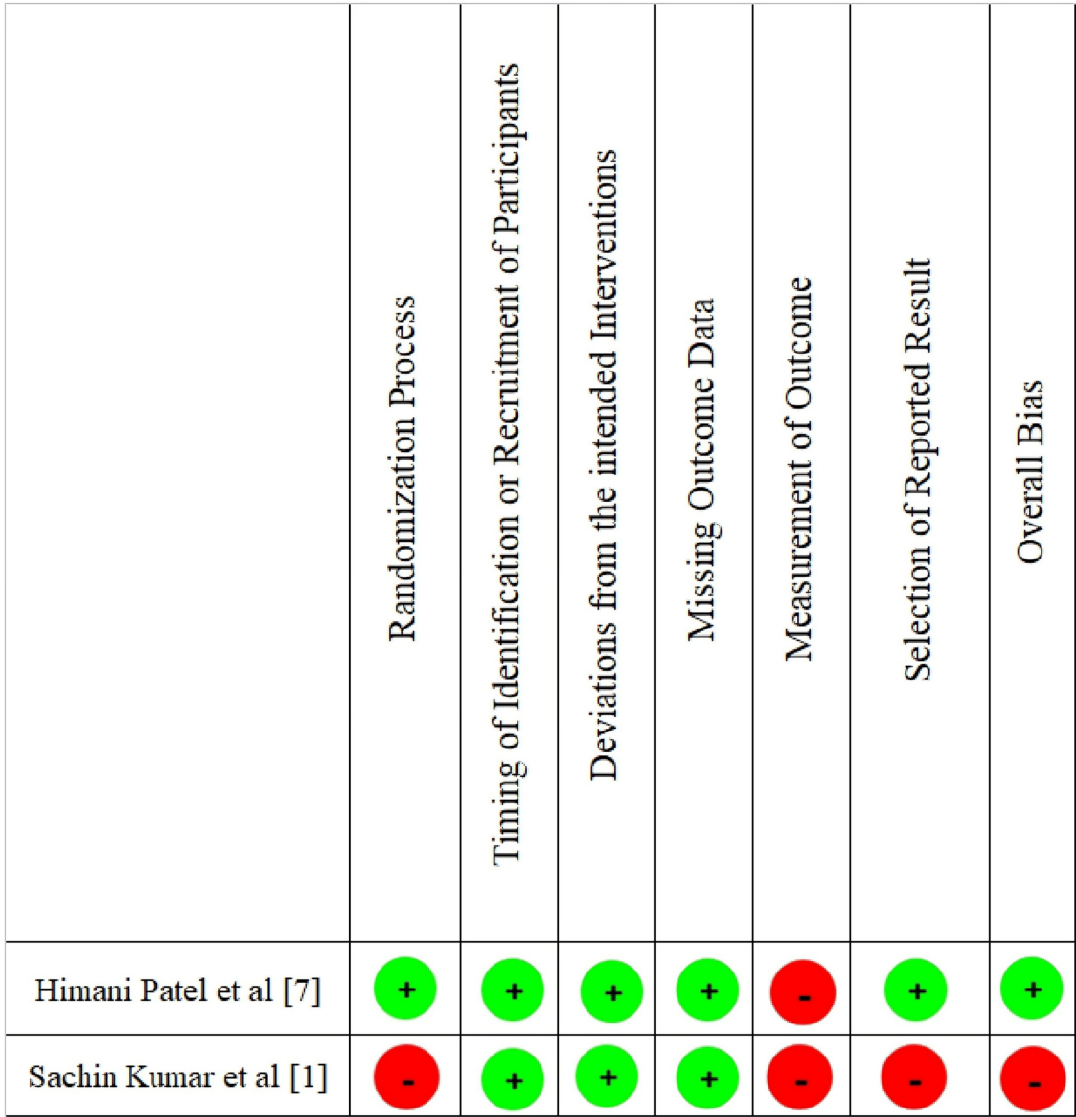
Summary of risk of bias for included randomized controlled trials (RoB-2)

**Figure 3:**
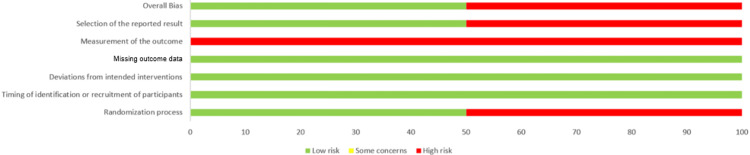
Risk of bias summary graph showing percentage distribution of judgments across RoB-2 domains

The quality of the included studies was assessed individually by the reviewers by using the RoB-2 tool for RCTs (Figures 2, 3) and the JBI checklist for other study designs (Tables 4-6).

**Table 4:** JBI questionnaire for risk of bias assessment in non-RCTs The revised JBI critical appraisal tool for the assessment of risk of bias in quasi-experimental studies [12] JBI: Joanna Briggs Institute; RCT: randomized controlled trial © JBI, 2020. All rights reserved. JBI grants use of these tools for research purposes only.

JBI questionnaire for non-RCTs	Singh et al., 2020 [6]
1. Is it clear in the study what the “cause” is and what the “effect” is (i.e., there is no confusion about which variable comes first)?	Yes
2. Was there a control group?	No
3. Were participants included in any comparisons similar?	Yes
4. Were the participants included in any comparisons receiving similar treatment/care, other than the exposure or intervention of interest?	No
5. Were there multiple measurements of the outcome, both pre and post the intervention/exposure?	No
6. Were the outcomes of participants included in any comparisons measured in the same way?	Yes
7. Were outcomes measured in a reliable way?	Yes
8. Was follow-up complete, and if not, were differences between groups in terms of their follow-up adequately described and analyzed?	Yes
9. Was appropriate statistical analysis used?	Yes
Overall score	66.60%

**Table 5:** Assessment of risk of bias in case series using the JBI questionnaire JBI: Joanna Briggs Institute Reference [13] © JBI, 2020. All rights reserved. JBI grants use of these tools for research purposes only.

JBI questionnaire for case series	Kanthan, 2016 [3]	Zhao et al., 2024 [5]	Hakim et al., 2019 [8]
				1. Were there clear criteria for inclusion in the case series?	Yes	No	Yes
2. Was the condition measured in a standard, reliable way for all participants included in the case series?	Unclear	Unclear	Unclear
3. Were valid methods used for identification of the condition for all participants included in the case series?	Yes	Yes	Yes
4. Did the case series have consecutive inclusion of participants?	No	Yes	No
5. Did the case series have complete inclusion of participants?	Yes	Yes	Yes
6. Was there clear reporting of the demographics of the participants in the study?	Yes	Yes	Yes
7. Was there clear reporting of clinical information of the participants?	Yes	Yes	Yes
8. Were the outcomes or follow-up results of cases clearly reported?	Yes	Yes	Yes
9. Was there clear reporting of the presenting site(s)/clinic(s) demographic information?	Yes	Yes	Yes
10. Was statistical analysis appropriate?	NA	NA	NA
Overall score	70%	70%	70%

**Table 6:** Assessment of risk of bias for case reports using the JBI questionnaire JBI: Joanna Briggs Institute JBI questionnaire for case reports [14] © JBI, 2020. All rights reserved. JBI grants use of these tools for research purposes only.

JBI questionnaire for case reports	Saripalli et al., 2022 [9]	Shteif et al., 2008 [10]	Peksöz et al., 2022 [11]
1. Were the patient’s demographic characteristics clearly described?	Yes	Yes	Yes
2. Was the patient’s history clearly described and presented as a timeline?	Yes	Yes	Yes
3. Was the current clinical condition of the patient on presentation clearly described?	Yes	Yes	Unclear
4. Were diagnostic tests or assessment methods and the results clearly described?	Yes	Yes	Yes
5. Was the intervention(s) or treatment procedure(s) clearly described?	Yes	Yes	Yes
6. Was the post-intervention clinical condition clearly described?	Yes	Unclear	Unclear
7. Were adverse events (harms) or unanticipated events identified and described?	NA	Unclear	Unclear
8. Does the case report provide takeaway lessons?	Yes	Yes	Yes
Overall score	87%	75%	62.50%

Quality ranking was allocated as low (less than 33%), medium (33%-66%), or high (over 67%). Out of nine studies, three studies were of medium quality [1,6,11], while the other six studies were of high quality [3,5,7-10]. Due to the lack of data and heterogeneity of included studies, a meta-analysis could not be performed.

Outcome Assessment

Pain assessment utilized the VAS across multiple studies, including those by Patel et al. [7], Kumar et al. [1], and Singh et al. [6]. Intraoperative fentanyl requirements were specifically measured in Patel et al.'s study [7], while postoperative rescue analgesic requirements were also evaluated in the same study. Intraoperative and postoperative hemodynamic monitoring, including changes in pulse rate, SpO_2_, and ECG parameters, was consistently assessed by Patel et al. [7], Kumar et al. [1], and Peksöz et al. [11]. Additional outcome parameters included the waiting period for initial analgesic demand and onset/duration of anesthesia, both measured in Kumar et al.'s study [1]. These varied assessment methodologies reflect the heterogeneity in outcome reporting across the included studies. It is further summarized in Table 7.

**Table 7:** Characteristics of outcomes SCPB: superficial cervical plexus block; GA: general anesthesia; VAS: Visual Analogue Scale; PONV: postoperative nausea and vomiting

Outcome measure	Studies reporting	Summary across studies
VAS pain scores (intra-/postoperative)	Patel et al. [7], Kumar et al. [1], Singh et al. [6]	Mean intraoperative VAS: 1.5 ± 1.87 (Singh et al.); postoperative VAS lower with SCPB vs. GA at 6 h (Patel et al., p = 0.005); duration-specific reductions (Kumar et al., p < 0.05 at multiple timepoints)
Intraoperative fentanyl requirement	Patel et al. [7]	Lower in the SCPB group (70.16 ± 13.09 μg) vs. GA (97.5 ± 13.75 μg, p = 0.001)
Postoperative rescue analgesics	Patel et al. [7], Kumar et al. [1]	Lower in SCPB: 833 ± 874 mg (Patel et al., p = 0.001); waiting period 137 ± 32 min (Kumar et al.) vs. 75 ± 14 min control (p = 0.031)
Hemodynamic stability (pulse, SpO_2_, ECG)	Patel et al. [7], Kumar et al. [1], Peksöz et al. [11]	Stable intra-/postoperatively across studies; no significant changes reported
Onset/duration of anesthesia	Kumar et al. [1]	Onset effective; duration extended postoperative analgesia
GA conversion rate	Singh et al. [6] (others N/R)	1/24 patients (4.2%) due to pain
Major complications	All 9 studies	None reported (0% across 140+ patients); minor PONV in GA control only (Patel et al.)

Discussion

This systematic review investigates the efficacy and safety of the SCPB as an alternative to GA in OMFS, specifically in procedures involving the mandibular and perimandibular regions. The increasing interest in regional anesthesia as a substitute for GA is driven by multiple factors: rising healthcare costs, the need for outpatient surgical options, and an aging population with significant comorbidities [15]. As such, this review addresses a clinically relevant question that has implications for patient safety, healthcare resource allocation, and the evolution of surgical anesthesia protocols in mandibular and perimandibular procedures.

The review included nine studies encompassing various research designs, including RCTs, prospective studies, case series, and case reports. Across the studies, 164 patients underwent mandibular and perimandibular surgical procedures using SCPB either as a sole anesthetic technique or in combination with local or regional nerve blocks. The most prominent finding was that SCPB consistently provided effective perioperative analgesia with minimal complications. 

Studies that included direct comparison with GA or local infiltration, such as those by Patel et al. [7] and Kumar et al. [1], demonstrated statistically significant improvements in intraoperative and early postoperative pain control in the SCPB groups. In the RCT by Patel et al., postoperative analgesic requirements and VAS scores at six hours were significantly lower in the SCPB group compared to GA. These results are indicative of SCPB's role in enhancing immediate postoperative comfort, potentially reducing reliance on opioid analgesics.

Similarly, Kumar et al. [1] found that patients receiving SCPB had an extended duration before requesting postoperative analgesia, suggesting better pain modulation. These studies lend credibility to the assertion that SCPB may enhance patient outcomes without the hemodynamic instability often associated with GA.

Case series and prospective clinical trials without control groups, such as those by Singh et al. [6], Kanthan [3], and Zhao et al. [5], further support SCPB's utility. They report high success rates and patient satisfaction, particularly in minor to moderate OMFS procedures such as abscess drainage, soft tissue lesion excision, and even mandibular fracture fixation when used adjunctively with other local blocks. Importantly, these studies did not report any major complications like nerve injury, systemic toxicity, or need for urgent conversion to GA (except one patient in Singh et al.'s study who reported unbearable pain).

GA remains the gold standard for extensive OMFS procedures, particularly those requiring absolute immobility, prolonged duration, or complex airway management. However, GA is not without drawbacks: cardiopulmonary complications, prolonged recovery time, postoperative nausea and vomiting (PONV) [16,17], and increased demand for perioperative resources. In contrast, SCPB offers several advantages: it avoids airway manipulation, maintains patient consciousness, and facilitates early ambulation and discharge. These attributes are particularly beneficial in patients with comorbidities or in outpatient surgical settings.

Moreover, the use of SCPB aligns with the principles of Enhanced Recovery After Surgery (ERAS), which emphasizes multimodal, opioid-sparing analgesia, early mobility, and minimal invasiveness [18-20]. By maintaining stable intraoperative vitals and offering superior postoperative pain control, SCPB contributes directly to these goals. As observed in the reviewed studies, none of the patients required airway support or reported severe PONV, common issues in GA [21,22].

A critical dimension of evaluating any anesthetic technique is its safety profile. SCPB is relatively safe, especially when performed using ultrasound guidance. Across the nine studies included in this review, the incidence of adverse effects was negligible. No major complications-such as phrenic nerve palsy, intravascular injection, or systemic toxicity-were reported. Only one study (Patel et al. [7]) noted two instances of PONV in the GA group, while none were reported in the SCPB cohort.

The superficial nature of SCPB reduces the likelihood of complications seen in deep cervical plexus blocks, such as epidural or subarachnoid spread. The block’s anatomical target, superficial sensory branches of C2-C4, limits motor involvement and minimizes the risk of respiratory compromise. These features make SCPB especially appealing for elderly or high-risk patients who might not tolerate GA well [23-25].

Limitations

Despite the encouraging findings, this review also highlights significant methodological limitations in the current literature, including small sample sizes and heterogeneity in study design that limit the generalizability of results. Only two studies used randomized control designs with blinding protocols, while others were observational or case-based with potential for selection and observer bias. Many studies did not use standardized tools for pain assessment, making inter-study comparisons difficult-some reported VAS scores, others did not quantify analgesic efficacy or relied on subjective feedback, and the lack of long-term follow-up data precludes assessment of sustained analgesic efficacy and delayed complications. Variations in SCPB technique (such as landmark-based versus ultrasound-guided), dosage of local anesthetics, and adjunctive block usage also contributed to heterogeneity, underscoring the need for a standardized protocol and uniform outcome measures to enable high-quality meta-analyses in future research. To date, no ERAS pathways explicitly integrating SCPB for OMFS procedures and no dedicated systematic reviews or meta-analyses of SCPB in OMFS have been reported. As a result, we relied on individual clinical studies of SCPB in OMFS together with broader literature on cervical plexus blocks, GA risk, and ERAS principles to frame our interpretation of SCPB-related outcomes.

## Conclusions

This systematic review affirms the efficacy and safety of the SCPB as a viable alternative to GA in selective oral and maxillofacial surgical procedures. SCPB provides comparable and, in many cases, superior intraoperative and immediate postoperative analgesia while maintaining a superior safety profile and patient satisfaction. Though more rigorous evidence is needed, current findings support broader incorporation of SCPB into anesthetic protocols, particularly in high-risk patients and resource-constrained environments. With standardization and further research, SCPB has the potential to become a cornerstone in regional anesthesia for OMFS.
